# Investigating the Relationship between Autistic Traits, Ruminative Thinking, and Suicidality in a Clinical Sample of Subjects with Bipolar Disorder and Borderline Personality Disorder

**DOI:** 10.3390/brainsci11050621

**Published:** 2021-05-12

**Authors:** Liliana Dell’Osso, Ivan Mirko Cremone, Giulia Amatori, Andrea Cappelli, Alessandro Cuomo, Stefano Barlati, Gabriele Massimetti, Antonio Vita, Andrea Fagiolini, Claudia Carmassi, Barbara Carpita

**Affiliations:** 1 Department of Clinical and Experimental Medicine, University of Pisa, 56126 Pisa, Italy; liliana.dellosso@med.unipi.it (L.D.); ivan.cremone@gmail.com (I.M.C.); giulia.amatori@libero.it (G.A.); andrea.cappelli1990@gmail.com (A.C.); gabriele.massimetti@med.unipi.it (G.M.); ccarmassi@gmail.com (C.C.); 2 Department of Molecular Medicine, School of Medicine, University of Siena, 53100 Siena, Italy; alessandrocuomo86@gmail.com (A.C.); andrea.fagiolini@unisi.it (A.F.); 3 Department of Clinical and Experimental Sciences, University of Brescia, 25123 Brescia, Italy; stefano.barlati@unibs.it (S.B.); antonio.vita@unibs.it (A.V.)

**Keywords:** autism spectrum, borderline personality disorder, bipolar disorder, suicide, autistic traits, autism spectrum disorder

## Abstract

(1) Background: Previous literature reported in both subjects with Borderline personality disorder (BPD) and Bipolar disorder (BD) higher levels of autistic traits, linked to a greater suicidality risk. The aim of this study was to evaluate and compare the presence of autistic traits in a sample of individuals with BD or BPD, with a specific focus on suicidality. (2) Methods: We recruited two clinical samples of subjects (BPD and BD) and a control group without a diagnosis according to DSM-5 (CTL). Subjects were assessed with the AdAS Spectrum, the RRS and, for evaluating suicidality, the MOODS-SR. (3) Results: The CTL group showed significantly lower scores of both BD and BPD on AdAS Spectrum, RRS, and suicidality scores. BPD subjects showed significantly lower scores than BD ones in most of AdAS Spectrum domain scores. Correlation and regression analyses highlighted specific patterns of association among AdAS Spectrum domains, RRS, and suicidality in each clinical group. (4) Conclusions: Both BPD and BD individuals show greater levels of autistic traits, which seem to be distributed in a continuum featuring the highest levels among BD subjects. In both disorders, higher autistic traits were linked to suicidal tendencies, although with different patterns of association between BD and BPD subjects.

## 1. Introduction

Autism Spectrum Disorder (ASD) is a neurodevelopmental condition characterized by persistent impairments in social communication and social relationships, narrow interests, repetitive behaviors and activities, with different grades of symptom severity [1]. ASD prevalence ranges from 0.76 to 2.64% in the general population, while prevalence rates in adult psychiatric inpatients vary from 2.4 to 9.9% across studies [2]. In the last decades, a wide number of research has highlighted how clinical ASD should be considered the extreme end of a continuum of behavioral and cognitive features, distributed in the clinical and general population, where they are often being labeled as “autistic traits” [3,4]. The concept of autistic traits, according to the most recent studies, refers to a range of milder manifestations, qualitatively similar to the core features of ASD (such as subthreshold social and communication impairments, unusual personality features and stereotyped behaviors), which were frequently reported among unaffected relatives of people with autistic conditions [5]. Autistic traits were firstly noticed and investigated among parents of children with ASD, leading to the conceptualization of a Broad Autism Phenotype (BAP) [6,7], and further stressing the presence of a continuum between clinical and subthreshold manifestations of the autism spectrum even from a genetic and neurobiological point of view [8,9,10,11,12]. On the other hand, autistic traits have been reported, as highlighted by several studies, not only in first-degree relatives of individuals with ASD, but also in other high-risk groups from the general population and in subjects affected by other psychiatric disorders [13,14,15,16,17,18,19,20,21]. Recent findings stressed the importance of recognizing and addressing autistic traits even with subthreshold due to their possible impact on quality of life [5]. In particular, while suicidal tendencies are known to be common in ASD, especially among individuals in the high-functioning end of the autism spectrum [16,22,23,24], contemporary literature is highlighting how the presence of suicidal thoughts and behaviors seems to also be more frequent among subjects with autistic traits in both general and clinical populations [13,16,17,20,25]. However, it should be noted that not all individuals with ASD or autistic traits reported suicidal ideation and behaviors. In particular, among ASD subjects, previous studies highlighted a prevalence rate for suicidal tendencies between 31 and 66% [23,26]. Pelton and Cassidy reported that in non-clinical samples, the presence of subthreshold autistic traits seem to be associated with suicidal behaviors. Specifically, the relationship between autistic traits and suicidal behaviors was mediated by perceived burdensomeness and thwarted belongingness [26].

Bipolar Disorder (BD) is a major affective disorder with a chronic course characterized by recurrent depression and mania/hypomania episodes. BD is one of the most severe psychiatric disorders that can be found in comorbidity with ASD. Together with unipolar depression and anxiety disorders, it is also one of the most common psychiatric disorders among ASD subjects [27]. The prevalence of BD in clinical samples of individuals with ASD ranges from 3 to 27% [28,29], while the prevalence of ASD in samples of subjects with BD ranges from 2 to 30%, depending on the study [30,31]. As observed in previous research, the presence of autism spectrum conditions in this population seems to be related to an earlier onset of BD, an increased number of affective episodes with mixed features and a greater functional impairment [32,33]. Intriguingly, in a sample of young adults with ASD, the presence of mood disorders was mediated by alexithymia and emotional deregulation [34]. Abu-Akel et al. [35] reported a 47.2% rate of subjects with significant levels of autistic traits in a sample of 797 BD subjects assessed with the Autism spectrum Quotient (AQ), short form. Moreover, significantly higher rates of anxiety disorders and significantly lower rates of substance use disorders were found in BD subjects with autistic traits [20]. While BD is also known to be associated with a high risk for suicidality, it has been reported that suicide ideation and behaviors may be markedly increased in individuals with both BD and ASD, and in particular among those with high-functioning ASD [36]. A recent study highlighted that, among subjects with BD, individuals with autistic traits show a higher suicidality and more severe depressive symptoms [20].

Another disorder with a well-known association with higher levels of self-injurious and suicidal behaviors is Borderline personality disorder (BPD) [37,38] BPD features a pattern of instability in different areas, especially in emotional regulation, interpersonal relationships, image of themselves, and impulse control [39]. BPD is a relevant clinical disorder associated with severe functional impairment and high rates of suicide, affecting 20% of psychiatric in-patients and 10% of out-patients [37,38]. As in the case of mood disorders, several studies highlighted the presence of an association between BPD and the autism spectrum. Some authors stressed how self-injurious behaviors may also occur among individuals with ASD, ranging from 47 to 56% and being more common among adolescents than in younger children [40]. According to Rydén et al., 15% of 41 females with BPD met ASD diagnostic criteria [41]. Another study, when comparing subjects with ASD, with BPD and controls, reported intermediate levels of autistic traits among BPD subjects when compared with the two other groups [42]. Nanchen et al. highlighted that about half of 38 BPD women scored beyond the cut-off on the AQ scale. In particular, BPD subjects with higher autistic traits showed lower scores of cognitive empathy and higher scores of alexithymia [43]. Conversely, Anckarsater et al. found a 12.2% prevalence of BPD in 74 individuals with ASD [44]. Another link between autism spectrum and BPD is the shared vulnerability towards traumatic events, which seems to lead in these subjects to higher rates of stress-related symptoms, eventually worsening the clinical picture [36,45,46,47]. A more recent study highlighted not only a higher presence of autistic traits among individuals with BPD, but also a link between autistic traits and greater suicidality risk in this population [16]. In a sample of 474 college students, other authors found a 17% rate of co-occurrence of autistic and borderline personality traits, also highlighting in this group the highest presence of suicidal ideation compared to students with only autistic or borderline personality traits [48].

The overlapping features between BD and BPD, in terms of mood deregulation, affective instability, impulsivity, suicidal ideation and behaviors, have been widely reported in the literature [49,50]. However, they are different clinical conditions not only from a psychopathological point of view, but also in terms of illness course, gender prevalence and heritability features [49,50]. Noticeably, as reported above, previous studies highlighted in both these populations a significant prevalence of autistic traits, which also seem to increase the suicidal risk in BD as well as in BPD subjects. On the other hand, autistic-like manifestations might have different characteristics, in terms of quality and/or quantity, among individuals with BD and BPD. These differences might also influence in specific ways other psychopathological features of the two disorders, including suicidal ideation and behaviors. In this framework, the aim of this study was to evaluate and compare the presence of autistic traits in a sample of individuals with BD or BPD, with a specific focus on how autistic traits may shape suicidal risk. A greater understanding of this matter may allow deepening the knowledge about the psychopathological core not only of BD and BPD, but also of the autism spectrum. Moreover, it may allow clinicians to develop better prevention, diagnostic and targeted therapeutic strategies for subjects with autistic traits and higher vulnerability factors in these clinical populations.

## 2. Materials and Methods

A consecutive sample of subjects with a clinical diagnosis of BD and of subjects with a clinical diagnosis of BPD were recruited at 3 Italian University Departments of Psychiatry (Pisa, Brescia and Siena). Moreover, a control group (CTL) was recruited by the same University departments. Exclusion criteria for all groups were: (I) Diagnosis of Schizophrenia or severe degenerative diseases; (II) language or intellectual impairment; (III) a current substance use disorder; (IV) age below 18 years or above 65 years. Inclusion criteria: (I) Subjects with a clinical diagnosis of BPD were included in the BPD group; (II) subjects who received a clinical diagnosis of BD I or II were included in the BD group; (III) subjects were included in the CTL group if they reported no current or lifetime mental disorders according to DSM-5 criteria. The study was conducted in accordance with the Declaration of Helsinki and the local ethics committee approved all the procedures. Written informed consent was obtained from all participants after they received a complete description of the study and had the opportunity to ask questions. All subjects were assessed with the following self-report instruments: The Adult Autism Subthreshold Spectrum (AdAS Spectrum), the Ruminative Response Scale (RRS) and the Mood Spectrum Self-report (MOODS-SR). 

### 2.1. The AdAS Spectrum

The Adult Autism Subthreshold Spectrum (AdAS Spectrum) was a self-report questionnaire developed with the aim of evaluating the wide range of symptoms and traits associated with the autism spectrum in adults without intellectual impairment. The questionnaire was composed of 160 items with dichotomous answers (yes/no), grouped in 7 domains. While the 1st domain (*Childhood/Adolescence)* investigates the presence of autistic-like features in the 1st decades of life, the other 6 domains evaluate the presence of symptoms and traits related to specific autism spectrum dimensions during the whole life-time (*Verbal communication*, *Non verbal communication*, *Empathy*, *Inflexibility and adherence to routine*, *Restricted interests and rumination*, *Hyper-Hypo reactivity to sensory input).* For each domain, a higher score indicates a higher presence of autistic-like features (e.g., greater difficulties in verbal and nonverbal communication, greater impairment of empathy skills, higher presence of inflexibility, restricted interests, ruminative thinking and hyper/hyporeactivity to sensory inputs). The AdAS Spectrum was developed simultaneously in English and Italian. The final Italian version showed excellent internal consistency and reliability, with a Kuder–Richardson’s coefficient of 0.96 for the total score and >0.80 for all the domains [15]. It has been proven to be in further studies a useful instrument for measuring autism spectrum in both clinical and non-clinical samples [5,15,16,17,18,19,20].

### 2.2. The RRS

The Ruminative Response Scale (RRS) was an instrument composed by 22 item rated on a 4-point Likert scale, tailored to assess the tendency to ruminative thinking. The scale was organized into 3 dimensions: *Brooding*, *reflection,* and *depression* and it has been often employed in previous literature for assessing the presence of rumination [51]. More recently, the instrument was developed and translated into Italian, showing good validity and reliability, with Cronbach’s alphas ranging from 0.77 and 0.80 [52].

### 2.3. The MOODS-SR

The MOODS, Self Report (MOODS-SR) questionnaire was an instrument tailored to evaluate the presence of a broad range of mood symptoms and temperamental traits during lifetime. It was composed of 160 items, which required a dichotomous answer (yes/no), grouped in 7 domains [53]. In keeping with previous studies [20], the MOODS-SR was employed for evaluating suicidality (suicidal ideation and behaviors), as measured by items 102 to 107 of the instrument. The MOODS was developed in both a structured clinical interview (SCI-MOODS) and a self-report form (MOODS-SR); for the Italian version the Cronbach’s alphas of the instrument ranged between 0.72 and 0.92, with a high agreement between the two formats (all intraclass correlation coefficients >0.88) [53].

### 2.4. Statistical Analyses

We performed chi-square and ANOVA analyses, followed by Bonferroni post-hoc tests, in order to compare demographic variables and scores reported on psychometric instruments between groups. Pearson’s correlation coefficient was used for evaluating the pattern of correlations between the scores reported on psychometric instruments within the 2 groups of BPD and BD subjects. Finally, in order to evaluate which AdAS Spectrum domains were statistically predictive of suicidality score in BD and in BDP subjects, 2 linear regression analyses (one for each diagnostic group) were performed (forward stepwise method) with suicidality total score as a dependent variable and AdAS Spectrum domains as independent variables. All statistical analyses were conducted using SPSS, version 20.

## 3. Results

The sample was composed of 58 BD subjects, 48 subjects with BPD and 59 CTL. Groups did not significantly differ for mean age, although significant differences were found in gender composition between groups (see Table 1). The CTL group showed significantly lower scores than both BD and BPD on all AdAS Spectrum and RRS scores, as well as on suicidality score. BPD subjects showed a significantly lower score than BD ones on all AdAS Spectrum domains, with the exception of AdAS Spectrum *Childhood/Adolescence*, on which BPD and BD groups did not report significant differences. No significant difference was found between individuals with BPD and BD when comparing scores reported on RRS and suicidality (see Table 1). In the BD group, suicidality score was significantly and positively correlated with all RRS domains and with AdAS Spectrum *Childhood/Adolescence*, *Nonverbal communication,* and *Restricted interests and rumination* domains (see Table 2). In the BPD group, the suicidality score showed significant and positive correlations with RRS *Reflection* and *Depression* domains, as well as with AdAS Spectrum *Nonverbal communication* and *Hyper-Hypo reactivity to sensory input* domain (see Table 3). Finally, results from linear regression analyses highlighted, in the BD group, a regression model featuring AdAS Spectrum *Restricted interests and rumination* as the only significant predictive variable of a higher suicidality score (beta = 0.40; t = 2.98; *p* = 0.005) (see Table 4). In the BPD group, while AdAS Spectrum *Nonverbal communication* and *Hyper-Hypo reactivity to sensory input* domain scores were identified as variables significantly and positively predictive of a higher Suicidality score (respectively beta = 0.42; t = 2.14; *p* = 0.038 and beta = 0.59; t = 3.02; *p* = 0.004), we also found that AdAS Spectrum *Inflexibility and adherence to routine* domain scores seemed to negatively predict a higher suicidality score (beta = −0.65; t = −2.97; *p* = 0.005) (see Table 5).

## 4. Discussion

The aim of the study was to assess the presence of autistic traits in a sample of subjects with BD or BPD, with a specific focus on which dimensions of the autism spectrum could represent predictive factors of suicidality. We found that autistic traits and rumination were more represented in both BD and BPD groups than in subjects without a diagnosis according to the DSM-5. These results are in line with previous studies that evaluated the prevalence of autistic traits among BD and BPD subjects. Matsuo et al. [33] found a 50% rate of autistic traits among 56 individuals with BD, by means of the Social Responsiveness Scale. Furthermore, a 7.2% rate of subjects with significant levels of autistic traits was observed by Abu-Akel et al. [35] in a sample of 797 individuals with BD assessed with the AQ short. More recently, Dell’Osso et al. [20] reported a 42.7% rate of autistic traits among subjects with BD. Regarding the relationship between autistic traits and BPD, results from previous studies showed that individuals with BPD have higher autistic traits than controls [16,41]. Moreover, Dell’Osso et al. [16] reported that autistic traits in individuals with BPD were associated with a history of physical or sexual abuse and to a greater suicidality risk. Comparing BD and BPD groups, our findings reported, in the BPD group, significantly lower scores on all AdAS Spectrum domains than in the BD one (with the exception of AdAS Spectrum *Childhood/Adolescence* domain, for which no statistically significant differences were found between the two groups). In particular, while BD subjects scored very close to 70 (specifically, 69.45 ± 27.12), which is the AdAS Spectrum threshold for the presence of ASD clinical symptoms, BPD subjects scored around 51, which is far below that threshold but still beyond the AdAS Spectrum threshold of 43, which marks the presence of relevant autistic traits [54]. These findings may lead to hypothesize a greater load of autism spectrum features and, possibly, of neurodevelopmental alteration severity, in BD than in BPD [55]. However, in line with previous studies in this field, our results highlighted higher ruminative thinking and suicidality tendencies in both the clinical groups than in the CTL group, without significant differences between BD and BPD subjects [16,20,36,37,38]. Suicidal thoughts seem to be very common among individuals with ASD [56,57]. Noticeably, a recent study highlighted that also subjects with autistic traits show an increased suicidality risk, without significant differences with respect to the risk reported among individuals with ASD [25]. Previous studies also reported higher levels of suicidal ideation among both BD and BPD subjects with comorbid autistic traits [16,20,36,41].

Our findings highlighted, in the two clinical groups, the presence of a specific pattern of features that seems to be predictive of suicidality. In the BD group, all RRS domains and AdAS Spectrum *Childhood/Adolescence*, *Non verbal communication* and *Restricted interests and rumination* domains, were significantly and positively correlated with suicidality scores. In the BPD group, significant correlations were reported between suicidality score and RRS *Reflection* and *Depression* domains, as well as with AdAS Spectrum *Non verbal communication* domain. However, when performing a regression analysis, the only significant predictive factor for suicidality among BD subjects was the presence of restrictive interests and rumination according to the AdAS Spectrum. On the other hand, in the BPD group AdAS Spectrum *Non verbal communication* and *Hyper-Hypo reactivity to sensory input* domain scores were identified as positive predictive factors of suicidality tendencies, while AdAS Spectrum *Inflexibility and adherence to routine* domain scores seemed to negatively predict a higher suicidality score. The presence of difficulties in non verbal communication among the autism dimensions more linked to suicidality, is in line with previous studies that hypothesized, in individuals with autistic traits, a link between the presence of a greater suicidal ideation and the presence of social and interpersonal difficulties, which would eventually lead to a higher risk of isolation and depressive symptoms [13,20,56]. The higher association found between suicidality and AdAS Spectrum *Non verbal communication* domain scores among BPD subjects may also be considered in line with previous findings that stressed how individuals in the mild range of the autism spectrum may be more aware of their difficulties in communicating with others, often developing social anxiety symptoms and maladaptive coping strategies, which may lead to more distress [55,58]. In particular, the high awareness of social communication difficulties seems to be particularly represented in female autism phenotypes, which often are characterized by the presence of camouflaging strategies [55,58]. Noticeably, both BPD and social anxiety are disorders more represented among females, and they feature, as the autism spectrum, an impairment of the social brain [59,60,61]. Ruminative thinking was another element strongly associated with suicidality, particularly among BD subjects. This finding is in line with a previous study that identified the same AdAS domain *Restricted interest and rumination* as a suicidality predictor [25]. Ruminative thinking is a core feature of ASD [25], and it may be more represented also among individuals with higher autistic traits. Rumination, as a negative pattern of repetitive thinking, often affects problem-solving and the ability to cope with negative feelings, exacerbating mood symptoms [25,62,63]. Among subjects with BPD we also found that the presence of inflexibility traits and adherence to a routine may be a protective factor towards suicidality tendencies. This intriguing data might be explained considering the high tendency towards loss of control and impulsive behaviors among individuals with BPD [45,64]. In this framework, it might be hypothesized that traits such as adherence to routine might work as compensatory factors with respect to the tendency towards emotional impulsivity, which was reported to be linked to suicidality risk among BPD subjects in previous studies [64]. 

This study should be considered in light of some obvious limitations. Firstly, the cross-sectional design of the study prevented us to evaluate possible temporal relationships between the considered variables. Moreover, the use of self-report instruments may have led to over- or underestimations of symptoms by the subjects, and to consequent biases in our results. Finally, the relatively small sample sizes, and the presence of differences in gender composition among groups, limit the extensibility of the present work. It should also be noted that, in the case of females, age is a major element that may affect mood regulation. Unfortunately, we were not able to assess in the clinical groups the presence of eventual mood alterations related to menopause among females with an age above 40 years. Although we did not find significant differences in mean age among groups, the comparatively lower mean age in the CTL group may have influenced our results. 

## 5. Conclusions

In the context of the above limitations, results from this study seem to globally highlight a significant presence of autistic traits in both BD and BPD samples, which seem to be distributed in a continuum with higher autistic traits in BD and lower in BPD subjects. In both disorders, higher autistic traits are linked to higher suicidal tendencies, confirming the high risk of suicide for this population. However, different patterns of association between autistic symptoms and suicidality were highlighted in the two disorders. In line with previous studies that stressed a possible neurodevelopmental basis for all psychiatric conditions, it might be hypothesized that different kinds of neurodevelopmental alterations may lead to different phenotypes of autism spectrum, in both quality and severity, shaping the different manifestations of psychiatric conditions [14,65]. Further studies should address the presence of specific patterns of autistic traits in different mental disorders, and how it might affect psychopathological trajectories and clinical outcomes.

## Figures and Tables

**Table 1:** Comparison of socio-demographic variables and psychometric instrument scores among groups.

	BD Group (*n* = 58)	BPD Group (*n* = 48)	CTL Group (*n* = 59)			
	*n* (%)	*n* (%)	*n* (%)	Chi-Square	*p*	Bonferroni’s Post-Hoc Comparisons (*p* < 0.05)
**Sex**				11.31	**0.003**	
M	37 (63.8)	15 (31.3)	27 (45.8)			M: BPD < BD;
F	21 (36.2)	33 (68.8)	32 (54.2)			F: BPD > BD
	**Mean ± SD**	**Mean ± SD**	**Mean ± SD**	**F**	***p***	
**Age**	35.48 ± 11.24	34.50 ± 9.67	32.86 ± 11.69	0.85	0.430	No significant differences
**AdAS Spectrum**						
Childhood/Adolescence	8.34 ± 4.20	7.10 ± 3.84	3.39 ± 2.51	30.36	**<0.001**	CTL < BD; CTL < BPD
Verbal communication	7.70 ± 4.18	5.73 ± 3.32	2.12 ± 1.98	43.10	**<0.001**	CTL < BPD < BD
Non verbal communication	12.04 ± 5.71	9.69 ± 5.19	4.59 ± 3.49	35.51	**<0.001**	CTL < BPD < BD
Empathy	5.20 ± 2.65	3.77 ± 2.76	1.71 ± 1.88	29.65	**<0.001**	CTL < BPD < BD
Inflexibility and adherence to routine	18.56 ± 7.88	12.72 ± 6.79	7.64 ± 5.50	37.09	**<0.001**	CTL < BPD < BD
Restricted interests and rumination	10.88 ± 4.16	8.08 ± 4.55	2.90 ± 2.89	61.13	**<0.001**	CTL < BPD < BD
Hyper-Hypo reactivity to sensory input	6.39 ± 3.83	4.15 ± 3.67	1.29 ± 1.46	38.66	**<0.001**	CTL < BPD < BD
AdAS Spectrum total score	69.45 ± 27.12	51.25 ± 24.01	23.64 ± 15.09	58.39	**<0.001**	CTL < BPD < BD
**RRS**						
Reflection	11.62 ± 3.04	11.36 ± 2.90	7.80 ± 2.98	28.95	**<0.001**	CTL < BD; CTL < BPD
Brooding	12.59 ± 3.01	13.30 ± 3.13	8.48 ± 2.46	45.15	**<0.001**	CTL < BD; CTL < BPD
Depression	31.13 ± 6.75	32.33 ± 8.06	19.34 ± 5.75	61.12	**<0.001**	CTL < BD; CTL < BPD
RRS total score	55.49 ± 11.15	57.11 ± 13.12	35.62 ± 9.82	61.01	**<0.001**	CTL < BD; CTL < BPD
**Suicidality (MOODS-SR)**	2.24 ± 2.02	2.230 ± 2.16	0.20 ± 0.78	26.06	**<0.001**	CTL < BD; CTL < BPD

**Table 2:** Pearson’s **c**orrelations coefficients (r) among Suicidality score and Adas Spectrum/RRS scores in the BPD group.

	Suicidality (MOODS-SR)
AdAS Spectrum total score	0.32 *
AdAS Childhood/Adolescence	0.35 *
AdAS Verbal communication	0.07
AdAS Non verbal communication	0.33 *
AdAS Empathy	0.15
AdAS Inflexibility and adherence to routine	0.20
AdAS Restricted interests and rumination	0.40 *
AdAS Hyper-Hypo reactivity to sensory input	0.24
RRS total score	0.65 *
RRS Reflection	0.56 *
RRS Brooding	0.48 *
RRS Depression	0.60 *

* Significant correlation for *p* <0.05.

**Table 3:** Pearson’s **c**orrelations coefficients (*r*) among Suicidality score and Adas Spectrum/RRS scores in the BPD group.

	Suicidality (MOODS-SR)
AdAS Spectrum total score	0.25 *
AdAS Childhood/Adolescence	0.03
AdAS Verbal communication	0.13
AdAS Non verbal communication	0.32 *
AdAS Empathy	0.11
AdAS Inflexibility and adherence to routine	0.12
AdAS Restricted interests and rumination	0.15
AdAS Hyper-Hypo reactivity to sensory input	0.28 *
RRS total score	0.37 *
RRS Reflection	0.32 *
RRS Brooding	0.26
RRS Depression	0.36 *

* Significant correlation for *p* <0.05.

**Table 4:** Stepwise linear regression with Suicidality score as a dependent variable and AdAS Spectrum domains as independent variables in the BD group.

	Variables	b (SE)	Beta	*t*	*p*	CI 95%	
						Upper Bound	Lower Bound
**Step 1**	Constant	0.69 (0.59)	-	1.16	0.250	−0.50	1.99
	AdAS Restricted interests and rumination	0.40	0.40	2.98	**0.005**	0.06	0.32

*R*^2^ = 0.161; adjusted *R*^2^ = 0.143.

**Table 5:** Stepwise linear regression with Suicidality score as a dependent variable and AdAS Spectrum domains as independent variables in the BPD group.

	Variables	B (SE)	Beta	*t*	*p*	CI 95%	
						Upper Bound	Lower Bound
**Step 1**	Constant	0.95 (0.53)	-	1.80	0.078	−0.11	2.00
	AdAS Hyper-Hypo reactivity to sensory input	0.21 (0.07)	0.40	3.00	**0.005**	0.07	0.35
**Step 2**	Constant	1.85 (.66)	-	2.79	**0.008**	0.51	3.18
	AdAS Hyper-Hypo reactivity to sensory input	0.37 (0.10)	0.71	3.61	**0.001**	0.16	0.58
	AdAS Inflexibility and adherence to routine	−0.10 (0.05)	−0.41	−2.11	**0.040**	−0.20	−0.01
**Step 3**	Constant	1.56 (0.65)	-	2.38	**0.021**	0.24	2.87
	AdAS Hyper-Hypo reactivity to sensory input	0.31 (0.10)	0.59	3.09	**0.004**	0.10	0.52
	AdAS Inflexibility and adherence to routine	−0.16 (0.05)	−0.65	−2.97	**0.005**	−0.27	−0.05
	AdAS Non verbal communication	0.15 (0.07)	0.42	2.14	**0.038**	0.01	0.29

*R*^2^ = 0.302; adjusted *R*^2^ = 0.256.

## Data Availability

Not applicable.
